# Expansions and contractions of the *FMR1* CGG repeat in 5,508 transmissions of normal, intermediate, and premutation alleles

**DOI:** 10.1002/ajmg.a.61165

**Published:** 2019-05-02

**Authors:** Sarah L. Nolin, Anne Glicksman, Nicole Tortora, Emily Allen, James Macpherson, Montserrat Mila, Angela M. Vianna‐Morgante, Stephanie L. Sherman, Carl Dobkin, Gary J. Latham, Andrew G. Hadd

**Affiliations:** ^1^ Department of Human Genetics New York State Institute for Basic Research in Developmental Disabilities Staten Island New York; ^2^ Department of Human Genetics Emory University School of Medicine Atlanta Georgia; ^3^ Wessex Regional Genetics Laboratory Salisbury NHS District Hospital Salisbury United Kingdom; ^4^ Biochemical and Molecular Genetics Hospital Clinic de Barcelona, IDIBAPS and CIBERER Barcelona Spain; ^5^ Department of Genetics and Evolutionary Biology, Institute of Biosciences Universidade de São Paulo São Paulo Brazil; ^6^ Asuragen, Inc. Austin Texas

**Keywords:** *FMR1*, fragile X, trinucleotide repeat instability

## Abstract

Instability of the *FMR1* repeat, commonly observed in transmissions of premutation alleles (55–200 repeats), is influenced by the size of the repeat, its internal structure and the sex of the transmitting parent. We assessed these three factors in unstable transmissions of 14/3,335 normal (~5 to 44 repeats), 54/293 intermediate (45–54 repeats), and 1561/1,880 premutation alleles. While most unstable transmissions led to expansions, contractions to smaller repeats were observed in all size classes. For normal alleles, instability was more frequent in paternal transmissions and in alleles with long 3′ uninterrupted repeat lengths. For premutation alleles, contractions also occurred more often in paternal than maternal transmissions and the frequency of paternal contractions increased linearly with repeat size. All paternal premutation allele contractions were transmitted as premutation alleles, but maternal premutation allele contractions were transmitted as premutation, intermediate, or normal alleles. The eight losses of AGG interruptions in the *FMR1* repeat occurred exclusively in contractions of maternal premutation alleles. We propose a refined model of *FMR1* repeat progression from normal to premutation size and suggest that most normal alleles without AGG interruptions are derived from contractions of maternal premutation alleles.

## 1 INTRODUCTION

*FMR1* is notable for instability of the cytosine guanine guanine (CGG) repeat in its 5′ untranslated region. This repeat can expand to more than 200 units (full mutation) which causes intellectual disabilities in males and some females by silencing the gene (Oberle et al., 1991; Verkerk et al., 1991; Yu et al., 1991). The repeat, which is highly polymorphic, is classified into four categories (Maddalena et al., 2001). Normal alleles (~5 to 44 repeats) are passed stably from parent to child with rare changes in repeat size. Intermediate alleles (45–54 repeats) occasionally undergo small changes in repeat size in some families during transmission whereas premutation alleles (55–200 repeats) are often highly unstable with large changes in repeat size from one generation to the next, which may result in full mutation expansions and the fragile X syndrome (FXS; OMIM 300624; Nolin et al., 2003). These categories correspond to the N (normal), S (high end normal, predisposed), Z (premutation), and L (full mutation) alleles in the population genetic model of the mutational process of the fragile X repeat (Morris et al., 1995; Morton & Macpherson, 1992).

Previous studies have identified three factors that contribute to repeat instability in *FMR1* premutation alleles: the sex of the transmitting parent, repeat size, and the presence of adenine guanine guanine (AGG) interruptions within the repeat. The first of these, parental sex, was recognized long before the gene was identified in 1991 (Sherman et al., 1985; Sherman, Morton, Jacobs, & Turner, 1984). Specifically, expansion to a full mutation occurs in maternal transmissions; virtually all premutation alleles from males are passed to daughters as premutation alleles although two rare examples of full mutation transmissions from fathers have been reported (Alvarez‐Mora et al., 2017; Zeesman et al., 2004). While males with full mutations generally do not have offspring, several reports and studies in sperm indicate the daughters will inherit premutations, not full mutations from their fathers (Reyniers et al., 1993; Willems et al., 1992). Females with full mutations have 50% risk of transmitting full mutations to their offspring who inherit an even greater repeat tract length (Nolin et al., 2003; Nolin, Ding, Houck, Brown, & Dobkin, 2008). The second factor, increased repeat size, increases the likelihood of expansion of a maternal premutation allele to a full mutation (Fu et al., 1991; Nolin et al., 2003). For paternal transmissions, the effect of repeat size has not been as well studied since the alleles are virtually always transmitted to the daughters as premutation alleles (Nolin et al., 1996; Sherman et al., 1984; Sherman et al., 1985). The third factor is the presence or absence of AGG triplets within the *FMR1* CGG repeat. Studies of primates and genetically distinct human populations indicate that the conserved repeat structure includes CGG repeats interspersed with regular AGG repeats (Eichler et al., 1995; Eichler & Nelson, 1996). In humans, the most common repeat structure of normal alleles includes an AGG as either the 10th or the 11th triplet and a second AGG after a stretch of nine more CGGs (Eichler et al., 1994; Gunter et al., 1998; Hirst, Grewal, & Davies, 1994; Kunst et al., 1996; Kunst & Warren, 1994; Snow, Tester, Kruckeberg, Schaid, & Thibodeau, 1994). Premutation alleles, in contrast, are less likely to contain AGGs and have long stretches of uninterrupted CGGs at their 3′ end. In 1994, Eichler et al. hypothesized that long tracts of uninterrupted CGGs in premutation alleles contribute to repeat instability and that tracts with more than ~34 uninterrupted CGGs can contribute to instability in smaller alleles as well. Recent studies (Nolin et al., 2013; Nolin et al., 2015; Yrigollen et al., 2012) have demonstrated that premutation alleles with no AGGs are at risk for expansion to full mutations in the next generation while alleles that include AGG interruptions are associated with greater intergenerational stability of the repeat.

In this study we surveyed more than 5,000 transmissions of normal, intermediate, and premutation alleles to examine the relationship of the sex of the transmitting parent, repeat size and pattern of AGG interruptions with allele instability. While repeat expansions and contractions were observed in all three size classes, repeat instability differed between maternal and paternal transmissions in each class. The distribution of different AGG configurations in the three size classes and in unstably transmitted alleles sheds light on the progression of *FMR1* alleles from normal to premutation repeat size over generations.

## 2 RESULTS

### 2.1 *FMR1* repeat structure in the general population

**Figure 1:**
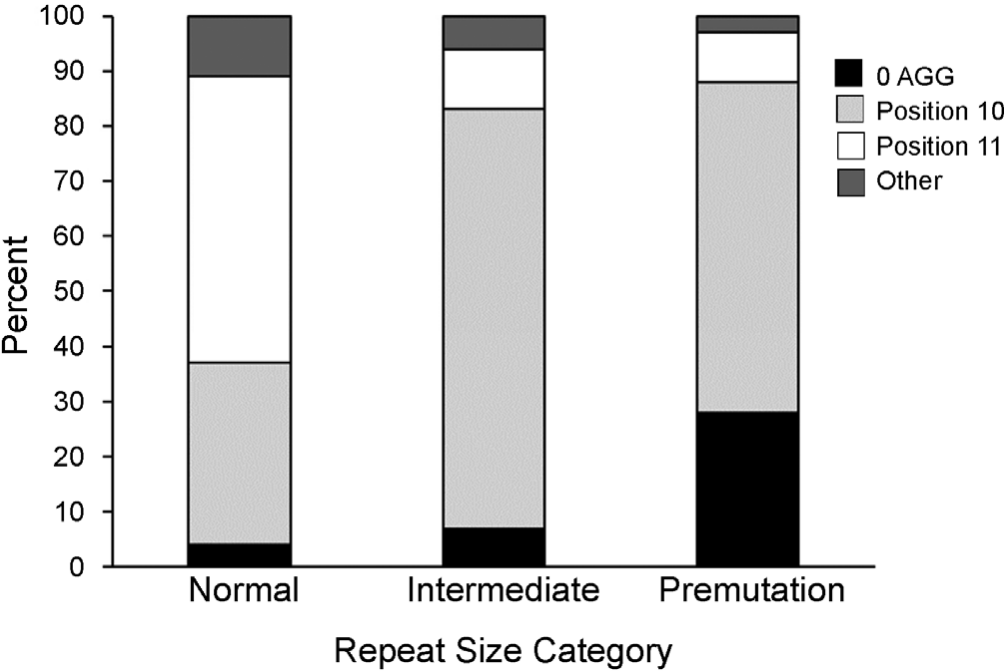
Repeat structure in normal, intermediate, and premutation alleles: presence or absence of AGG interruptions and position of the first AGG triplet within the repeat

We first examined the repeat structure of 5,623 normal alleles, 406 intermediate alleles and 1,574 premutation alleles. The six most common normal alleles comprising 67.6% of alleles in the general population are shown in Table 1. Alleles with 30 repeats and the structure (CGG)_10_AGG(CGG)_9_AGG(CGG)_9_ (represented as 10A9A9) were the most frequent (33.9%) followed by 29 repeat alleles (17.4%) with the structure 9A9A9. Eighty‐five percent of normal alleles had nine or ten uninterrupted repeats at the 3′ end while 12.4% (699/5,623) had more than 18 uninterrupted repeats. To compare repeat structures in the different size classes, we examined the presence or absence of AGG as well as the position of the first AGG within the repeat (Figure 1). First, there was an increase in the number of alleles without AGGs as the size class increased. Among normal alleles, 4.2% (234/5,623) contained no AGG interruption compared to 7.4% (30/406) of intermediate alleles and 27.9% (439/1,574) of premutation alleles (*χ* ^2^ w 2 df; *p* < .0001). Second, as the repeat size lengthened, the proportion of alleles with an AGG as the 10th triplet increased while alleles with an AGG as the 11th triplet decreased. Among normal alleles, 32.6% (1,833/5,623) had an AGG as the 10th triplet and 52.5% (2,951/5,623) as the 11th triplet. In contrast, 75.6% (307/406) of intermediate alleles had an AGG as the 10th triplet and 11.3% (46/406) as the 11th triplet. For premutation alleles, 59.7% (939/1,574) had an AGG at the 10th triplet and 9.2% (145/1,574) at the 11th triplet (*χ* ^2^ w 2 df; *p* < .0001).

**Table 1:** Structure and frequency of the six most common normal alleles identified among 5,623 alleles obtained from this pan‐ethnic study sample

Repeat tract length	Repeat structure^a^	Frequency
20	10A9	0.062
23	13A9	0.031
29	9A9A9	0.174
30	10A9A9	0.339
31	10A9A10	0.049
32	9A12A9	0.021

a Numbers represent the number of CGG triplets; A represents an AGG interruption.

### 2.2 Instability of normal, intermediate, and premutation alleles on transmission

We analyzed expansions and contractions of the *FMR1* repeat in transmissions of 3,335 normal, 293 intermediate and 1,880 premutation alleles (Table 2). The 14 (0.4%) unstable transmissions of normal alleles identified among 2,020 maternal and 1,315 paternal transmissions are listed in Table 3. Four maternal normal alleles (0.19%) and 10 paternal normal alleles (0.76%) exhibited instability, all four of the maternal alleles and seven of the 10 paternal alleles increasing by a single repeat. One paternal allele increased by two repeats and another by five repeats. One contraction was observed in a paternal allele that underwent a two‐repeat reduction in size. Two paternal alleles with 44 repeats each gained one repeat and in the following generation were categorized as intermediate alleles. The smallest and largest unstable normal alleles had 27 and 44 repeats respectively, each with no AGG interruptions. All of the unstable normal alleles contained more than 18 uninterrupted CGG repeats at the 3′ end compared to only 12.4% (699/5,623) of normal alleles. Six alleles (42.9%) contained no AGG interruptions compared to 4.2% in the general population (Fisher exact test *p* < .01). Six alleles contained one AGG interruption and only two contained two AGG interruptions. Where the presence of AGG interruptions established polarity, all changes occurred on the 3′ end. No loss of AGG interruptions was observed in 1021 maternal and 537 paternal transmissions of normal alleles whose AGG structure was known.

**Table 2:** Instability of normal, intermediate, and premutation alleles on transmission

	Maternal			Paternal		
Parental repeat tract length^a^	Expand (%)	Contract (%)	Total	Expand (%)	Contract (%)	Total
<45	4 (0.2)	0	2,020	9 (0.7)	1 (0.08)	1,315
45–54	37 (14.8)	5 (2.0)	250	12 (27.9)	0 (0)	43
55–64	324 (54.5)	11 (1.8)	595	45 (68.2)	3 (4.5)	66
65–74	238 (86.9)	12 (4.4)	274	42 (79.2)	5 (9.4)	53
75–84	195 (92.9)	15 (7.1)	210	58 (72.5)	21 (26.3)	80
85–94	106 (94.6)	5 (4.5)	112	27 (58.7)	17 (37.0)	46
≥95	318 (98.2)	5 (1.5)	324	29 (24.2)	85 (70.8)	120
Total	1,222 (32.3)	53 (1.4)	3,785	222 (12.9)	132 (7.6)	1,723

a <45 normal, 45–54 intermediate, and ≥55 premutation.

**Table 3:** Transmissions of unstable normal alleles

Origin	Parental repeat track length	Parental repeat structure^a^	Offspring repeat structure	Change in repeat size
Maternal	28	28	29	+1
Maternal	33	33	34	+1
Maternal	39	9A29	9A40	+1
Maternal	41	41	42	+1
Paternal	27	27	28	+1
Paternal	29	9A19	9A20	+1
Paternal	33	10A22	10A24	+2
Paternal	34	34	35 & 35^b^	+1
Paternal	35	9A25	9A30	+5
Paternal	39	9A9A19	9A9A20	+1
Paternal	40	9A30	9A31	+1
Paternal	41	9A31	9A29	−2
Paternal	44	9A9A24	9A9A25	+1
Paternal	44	44	45	+1

a Numbers represent the number of CGG triplets; A represents an AGG interruption.

b Twin daughters 70% likelihood of dizygosity.

There were 250 maternal and 43 paternal transmissions of intermediate alleles in this survey (Table 2). Paternal intermediate alleles were nearly twice (27.9%) as likely to expand as maternal alleles (14.8%) (two tailed Fisher exact test; *p* = .04). There were five contractions of maternal intermediate alleles and no contractions of paternal alleles. The five maternal contractions resulted in one normal allele and four intermediate alleles. No loss of AGGs was observed in the 287 transmissions of intermediate alleles with AGG interruptions.

There were 1,515 maternal and 365 paternal transmissions of premutation alleles. Expansions occurred in 77.9% (1,181/1515) of the maternal transmissions and in 55.1% (201/365) of paternal transmissions (Table 2). Unstable transmissions of paternal and maternal alleles occurred with increasing frequency as the parental repeat size increased. There were, however, striking differences between the patterns of maternal and paternal instability. In maternal transmissions the frequency of expansions increased continuously with repeat size while in paternal transmissions the frequency of contractions increased continuously with repeat size (from 4.5% to more than 70%). In total, 35.9% (131/365) of paternally transmitted alleles underwent contractions in size, all resulting in premutation alleles in the next generation. In contrast, contractions occurred in only 3.2% (48/1515) of maternal transmissions and resulted in six (12.5%) normal, 13 (27.1%) intermediate, and 29 (60.4%) premutation alleles. Maternal alleles with no AGGs (6.0%) were more likely to contract than those with one (3.4%) (Fisher exact test *p* = .06) or two AGGs (2.1%) (Fisher exact test *p* < .01). No significant effect of AGG interruptions on contractions was detected in paternal transmissions (Table 4).

**Table 4:** Parental AGG number and repeat contractions on transmission

	Maternal		Paternal	
No. AGGs	Contractions/transmissions	%	Contractions/transmissions	%
0	19/316	6.0	56/163	34.3
1	22/650	3.4	53/156	34.0
2	10/484	2.1	15/68	22.1
Total	51/1450	3.5	124/387	32.0

Examination of the contractions of five intermediate and 48 premutation maternal alleles revealed that contractions occurred more frequently for alleles with 65–94 repeats (5.37%; 32/596) compared to other intermediate and premutation alleles (1.94%; 21/1169) (two tailed Fisher exact test *p* = .001). Multiple contractions from four mothers were observed (Table 5). Two mothers carrying 79 repeat alleles had two transmissions with contractions, and a third mother with 79 repeat alleles had contractions in two of three children that inherited the expanded allele. A fourth mother with an 83 repeat allele had three transmissions with contractions and a fourth transmission that expanded. In each case, the contractions resulted in smaller premutations that were similar in size within each family and with no AGGs lost. We calculated the likelihood of observing multiple contractions from these four mothers, assuming that each contraction within a family was an independent event. We estimated the probability of a contraction from women in the sample with 65–94 repeat alleles who had only one transmission and that underwent a contraction (i.e., *p* = 18/354 = .051; 95% confidence interval = 0.028–0.074). The likelihood estimates of the four observations are outlined in Table 5 and are based on a binomial distribution for contractions. This analysis indicated that the number of contractions observed in these four mothers would not be expected to occur in this study population if contractions were independent events.

**Table 5:** Comparison of the observed and expected number of mothers with multiple contractions of premutation alleles

Multiple contractions (*n* contractions/*n* transmissions)	2/2		2/3	3/4
Repeat structure in 4 mothers	10A68, 9A69		79	83
Likelihood of observed contractions	*p* ^2^		3*p ^2^*(1 − *p*)	4*p ^3^*(1 − p)
Estimated likelihood assuming independence of events (*p* = .0508 or .0737^a^)	0.003, 0.006		0.007, 0.015	0.0005, 0.002
Number of mothers with 2, 3, or 4 transmissions	85		15	5
Expected number of mothers with multiple contractions (*p* = .0508, .0737)	0.22, 0.47		0.11, 0.23	0.002, 0.01
Observed number of mothers with multiple contractions	2		1	1

a 0.0508 is the empirical estimate of the frequency of repeat contractions based on mothers having only one transmission (see text); 0.0737 is the upper limit of the 95% confidence interval of this estimate.

### 2.3 AGG loss on transmission

The number of AGG interruptions in intermediate and premutation repeats was determined for transmissions of 1,450 maternal and 387 paternal alleles (Table 4). The loss of AGG interruptions is summarized in Table 6 for the 32 maternal and 68 paternal premutation alleles with AGGs that contracted during transmission. Eight maternal alleles lost AGGs in contractions whereas no AGG loss was observed in paternal contractions, a highly significant difference (two‐tailed Fisher exact test *p* = .0002). Seven maternal alleles lost one AGG and an eighth lost two AGGs (Table 7). These eight alleles had their first AGG at position 10. These maternal contractions resulted in three normal, two intermediate and three smaller premutation alleles. While information about AGG interruptions was not available for all transmissions, no AGGs were lost in expansions of 415 maternal and 109 paternal premutation transmissions.

**Table 6:** Number of contractions in which the AGG interruption was lost during transmission of a premutation allele

	Maternal	Paternal
Contractions	32	68
AGG loss	8	0

**Table 7:** Repeat structure of maternal alleles that lost AGGs during transmission

Maternal allele^a^	Offspring allele	No. AGGs lost
9A45^b^	16	1
9A9A42	45	2
9A64	20	1
9A67	59	1
9A68	61	1
9A69	47	1
9A9A75	9A24	1
9A118	84	1

a Numbers represent the number of CGG triplets; A represents an AGG interruption.

b In this family the grandfather carried a 29 repeat allele and the grandmother a 55 repeat allele. The mother was mosaic for 16, 29, and 55 repeat alleles and the grandson inherited a 16 repeat allele.

## 3 DISCUSSION

In this study of more than 5,000 transmissions of normal, intermediate, and premutation alleles, we observed expansions and contractions in all three size classes. This instability was strongly influenced by the sex of the transmitting parent and by the repeat structure—number of repeats and location of the AGG interruptions—in the parental allele. The results of this survey suggest that instability is intrinsic to all repeat alleles and the frequency of these events is a continuous function influenced by repeat structure and parental sex.

Expansions of maternal premutation alleles on transmission have been well documented in various studies since the *FMR1* gene was identified in 1991 (Fu et al., 1991; Nolin et al., 2003; Snow et al., 1994). These expansions are understood to occur by additions to the 3′ uninterrupted CGG repeat and the AGG configuration at the 5′ end of the repeat is unlikely to be changed. Contractions of premutation alleles, however, are uncommon and have received less attention. We identified three striking differences between contractions of maternally and paternally transmitted alleles. First, among premutation‐sized alleles, contractions in paternal transmissions were common and occurred 10 times as frequently (35.9%) as in maternal transmissions (3.2%). The frequency of paternal contractions increased linearly with repeat size which may be due to increased mitotic instability of large repeats in spermatogonia prior to meiosis (Ashley‐Koch et al., 1998; Nolin et al., 1999). Although the patterns of instability differ, studies in the fragile X mouse model support the idea of instability in spermatogonia (Zhao & Usdin, 2018). Maternal contractions appear to peak at 80 repeats which likely results from the increasing frequency of full mutation expansions in maternal transmissions of larger repeat alleles. Second, each of the contractions transmitted from premutation fathers resulted in smaller alleles that were, without exception, still in the premutation size range. In contrast, maternal contractions resulted in normal or intermediate alleles in 40% of transmissions. Thus, in maternal transmissions, the magnitude of repeat size change extends not only to expansions but to contractions as well. Third, the loss of AGG interruptions was observed only in contractions of maternal premutation alleles. While maternal contractions were infrequent, loss of an AGG interruption in these contractions was common and occurred in 25.0% (8/32) of maternal alleles with AGGs. This is an unexpectedly high frequency particularly considering that 68 contractions of paternal premutation alleles were not associated with any AGG loss in transmission. The longest pure repeat associated with AGG loss was 118 CGGs. The mechanism of maternal contractions appears to be complex as a maternal allele with 9A9A42 was reduced to 45 CGGs and another maternal allele with 9A9A75 was transmitted to the offspring as 9A24. We observed no AGG loss in expansions or contractions of paternal alleles although there is one report of a paternal 52 repeat allele with two AGGs that was transmitted to a daughter as an expanded 56 repeat allele with no AGGs (Fernandez‐Carvajal et al., 2009). Our study indicates that changes in AGG interruptions are rare aside from infrequent loss of AGG interruptions in transmission from parent to child. Thus, it is likely that some full mutation expansions include AGG interruptions. In contrast to the general stability of AGG interruptions in the *FMR1* repeat, intergenerational variations in location of repeat interruptions in myotonic dystrophy type 1 are not uncommon (Musova et al., 2009).

The importance of AGG interruptions in maintaining *FMR1* repeat stability has been well documented in studies of human transmissions (Nolin et al., 2013; Nolin et al., 2015; Yrigollen et al., 2012), but has not shed light on the mechanisms. Studies in yeast (Rolfsmeier & Lahue, 2000) have shown that the presence of AGG interruptions prevent the formation of hairpin intermediaries. Other studies (Jarem, Huckaby, & Delaney, 2010; Pearson et al., 1998) have shown DNA conformational differences in repeat length tracts with and without AGG interruptions. In addition, tracts with pure repeats have been associated with altered nucleosome assembly (Bettencourt et al., 2016; Genetic Modifiers of Huntington's Disease Consortium, 2015; Moss et al., 2017; Tome et al., 2013; Volle & Delaney, 2013).

Among the mothers whose premutation alleles contracted in transmission, there were four with more than one contraction. Likelihood analyses showed that their occurrence was unlikely, assuming independence of the probability of a contraction, suggesting that genetic factors might have a role. Because there were three different haplogroups (data not shown) represented in these families, it is unlikely that cis factors had a role in these contractions. Furthermore, sisters in two of the families did not transmit contracted alleles. A series of DNA repair genes including *MLH1*, *MSH3*, *FAN1*, and *PMS2* have been implicated in the repeat instability observed in different repeat disorders (Bettencourt et al., 2016; Genetic Modifiers of Huntington's Disease Consortium, 2015; Moss et al., 2017; Tome et al., 2013). Studies in the fragile X mouse model have shown that the mismatch repair proteins MSH2 and MSH3 in the MutSβ complex have a role in contractions as well as repeat expansions (Zhao et al., 2015). Thus, polymorphisms in DNA repair proteins may affect the likelihood of contractions.

The 14 unstable transmissions of normal size alleles illustrate the importance of overall allele length, structure, and parental origin as determinants of instability. All these unstable normal alleles contain more than 18 uninterrupted 3′ CGGs repeats compared to only 12.4% of normal alleles. Another structural feature, the absence of AGGs, is also a marker of instability. In our survey, 6 of the 14 unstable normal alleles contained no AGGs which was approximately 10 times the frequency we observed among normal alleles overall. Thus, similar to the much larger premutation alleles, normal alleles lacking AGGs have a greater likelihood of intergenerational repeat instability. For alleles that contain AGGs, the position of the AGG appears to be important in maintaining stability on transmission. Although 52.5% of normal alleles had an AGG as the 11th triplet, only one of the seven (14.3%) unstable normal alleles with AGGs has an interruption at that position. The other six (85.7%) had an AGG at position 10 compared to only 32.6% of normal alleles. Furthermore, the proportion of repeats with AGGs at the 10th position increases from 32.6% in normal alleles to 75.6% in intermediate alleles. These observations suggest that alleles with an AGG at position 10 may be more unstable than those with an AGG at position 11. Other investigators have also observed an excess of AGGs at position 10 associated with intermediate and premutation alleles (Eichler et al., 1995; Gunter et al., 1998; Kunst & Warren, 1994; Murray et al., 1997; Snow et al., 1994).

The sex of the transmitting parent has an impact on stability for normal, intermediate, premutation, and full mutation alleles. An earlier study by Sullivan et al. (2002) showed greater instability of normal and intermediate alleles in paternal than maternal transmissions. In our survey, unstable transmissions of normal paternal alleles (0.76% [10/1,315]) occurred more than three times as frequently as maternal (0.2% [4/2,020]) (Fisher exact: *p* < .05). For intermediate alleles, 27.9% (12/43) of paternal alleles were unstable compared to 16.8% (42/250) of maternal alleles (Fisher exact: *p* < 0.1). Thus, although unstable maternal transmissions receive the most attention because of risk of full mutation expansions, unstable paternal transmissions are more frequent for normal and intermediate alleles. This is consistent with the higher mutation rates for sperm (Crow, 2000). Males and females with full mutations also have very different risks for transmitting full mutation alleles to their offspring. Females with full mutations have 50% risk with each pregnancy of passing full mutation alleles to the next generation that inherit larger full mutation alleles than their mothers (Nolin et al., 2008). Most males with full mutations do not have offspring, but rare reports of such events indicate that the daughters inherited premutation alleles from their fathers (Willems et al., 1992). Furthermore, analysis of sperm from full mutation males revealed only premutation alleles (Reyniers et al., 1993) confirming the instability of full mutation repeats in the male germline. The different outcomes of *FMR1* full mutation allele transmissions from males and females result from differences in gametogenesis. In both sexes, the gametes segregate as primordial germ cells between days 5 and 12 after fertilization. With sexual maturity in males, the germ cells undergo numerous mitotic divisions with perhaps as many as 549 divisions (Vogel & Rathenberg, 1975) leading to contractions of the long repeat tracts as a consequence of repair associated with genome maintenance and DNA replication before entering into meiosis (Pearson, 2003). This idea is consistent with our study showing that contractions occur with greater frequency in transmissions of males with larger premutation repeats. In contrast, in females, oogonia undergo a limited number of mitotic divisions before entering into meiosis I as primary oocytes where meiosis is halted until puberty when ovulation occurs with completion of meiosis II after fertilization. This extended period in meiosis I may lead to the formation of alternative DNA structures that cannot be easily repaired on completion of meiosis and result in full mutation expansions. Studies of female fetuses with full mutations suggested that methylation patterns had not been established in oocytes of the 16‐week‐old female fetus. In a 17‐week‐old male fetus with a full mutation immunohistochemistry of testis detected *FMR1* protein in some cells which suggests that contractions to premutation tract lengths had occurred (Malter et al., 1997).

**Figure 2:**
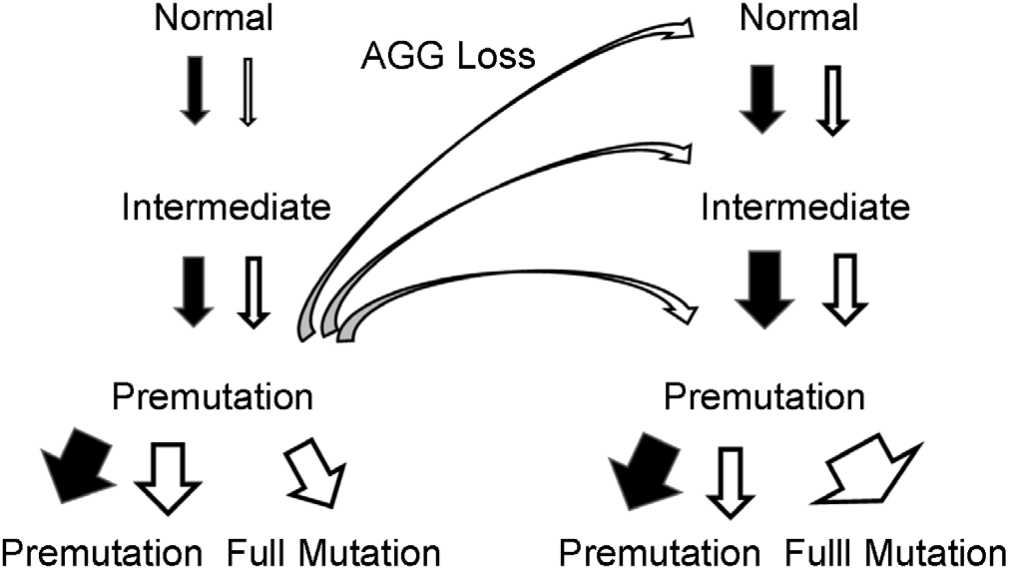
Model with two pathways for fragile X repeat expansion. The black arrows represent transmissions from males and the white arrows transmissions from females. The curved white arrows represent loss of AGGs in contractions of maternal premutation alleles. The width of the arrows indicates greater or less instability on transmission

Our study supports and refines the model of fragile X population genetics originally proposed by Morton and Macpherson (1992), Morris et al. (1995) and modified by Eichler et al. (1996) who suggested there are two pathways for expansion of *FMR1* alleles. Our analysis of 5,508 transmissions suggests that the generation of unstable premutation alleles and their expansion to a full mutation is a multi‐step process that often includes contractions of maternal premutation alleles to smaller alleles without AGGs. We propose a refinement of the two‐pathway model for repeat expansion (Figure 2). The first path is slow with a gradual accumulation of CGG repeats in normal alleles containing AGG interruptions. Small increases are more likely to occur in paternal than maternal transmissions of normal and intermediate alleles. Alleles with an AGG at the 10th position within the repeat are also more likely to increase in size than those with an AGG at position 11. Rare increases of a cassette of 10 repeats from 29 to 39 repeats with the final structure (CGG)_9_AGG(CGG)_9_AGG(CGG)_9_AGG(CGG)_9_ in a single transmission (Macpherson et al., 1995) may also result in repeat size increases. In support of this idea, 11/16 alleles with 39 repeats in our survey have this structure with three AGG interruptions each separated by nine CGGs. Through various mechanisms over numerous generations, normal alleles may reach premutation size with ≥80 repeats and become highly unstable. A second path for expansion is suggested by the AGG loss observed in our studies. Premutation alleles may lose AGGs through contractions and become normal, intermediate, or smaller premutation alleles. We show here that normal alleles without AGGs are disproportionately likely to gain repeats as compared to those with AGGs as suggested by earlier studies with sperm small pool PCR. (Crawford, Wilson, & Sherman, 2000) Thus, these normal or intermediate alleles without AGGs are prone to more rapid intergenerational expansions to premutation size. Small premutation alleles without AGGs then have a high likelihood to expand rapidly to full mutations within a generation or two. These findings suggest contractions of maternal premutation alleles are a major mechanism for generating new alleles without AGG interruptions that are at high risk for eventual expansion to full mutation alleles.

## 4 MATERIALS AND METHODS

### 4.1 Ethical policies and ethical considerations

The samples used in the study were discarded clinical specimens.

### 4.2 Subjects

To examine the repeat structure distribution of normal alleles in the general population, alleles from one person in each pedigree from our database as well as partners marrying into a family were included for a random sampling of alleles. Forty‐six percent of the ethnicities were known: 67% Caucasian, 13% Hispanic, 8% African‐American, 7% Asian, 3% mixed race, and 2% other. DNA was isolated from leukocytes or for prenatal samples from chorionic villi or amniocytes.

The 2020 maternal transmissions of normal alleles from 1,533 mothers were from 147 who carried two normal alleles, 179 with an intermediate allele, 1,116 with premutation alleles, 89 with full mutation alleles, and two mothers who carried deletions within the *FMR1* gene. The 1,765 maternal transmissions of intermediate and premutation alleles were from 210 mothers who carried an intermediate allele and 1,059 mothers with a premutation allele. The 1,315 paternal transmissions of normal alleles were from 1,048 fathers who were members of fragile X families. The 43 paternal transmissions of intermediate alleles were from 29 fathers and the 365 paternal transmissions of premutation alleles were from 203 fathers with a premutation and 11 unaffected fathers with full mutation alleles who transmitted premutation alleles to their daughters.

### 4.3 PCR protocols

The *FMR1* repeat sizing was performed by PCR analysis using AmplideX PCR CE/*FMR1* (Asuragen, Inc.) and the products separated by capillary electrophoresis with Applied Biosystems 3130. The AGG interruption patterns were determined using AmplideX PCR CE/*FMR1* at the New York State Institute for Basic Research in Developmental Disabilities or Xpansion Interpreter (Asuragen, Inc.) at Asuragen. All transmissions were categorized by parental sex. Changes in allele repeat length on transmission were determined by comparison of parental and transmitted alleles in parallel PCR capillary electrophoretic analyses. An unstable transmission was defined as a change of at least one repeat from parent to child.

## CONFLICT OF INTERESTS

Gary Latham is a full‐time employee of Asuragen, Inc. He and Andrew Hadd have stock options in Asurgen, Inc. The other authors declare no competing interests.
